# Bridging metabolic syndrome and cognitive dysfunction: role of astrocytes

**DOI:** 10.3389/fendo.2024.1393253

**Published:** 2024-05-10

**Authors:** Zihan Li, Ya-yi Jiang, Caiyi Long, Xi Peng, Jiajing Tao, Yueheng Pu, Rensong Yue

**Affiliations:** ^1^ Department of Endocrinology, Hospital of Chengdu University of Traditional Chinese Medicine, Chengdu, China; ^2^ Clinical Medical School, Chengdu University of Traditional Chinese Medicine, Chengdu, China

**Keywords:** metabolic syndrome, cognitive dysfunction, astrocytes, immunometabolism, neurodegeneration

## Abstract

Metabolic syndrome (MetS) and cognitive dysfunction pose significant challenges to global health and the economy. Systemic inflammation, endocrine disruption, and autoregulatory impairment drive neurodegeneration and microcirculatory damage in MetS. Due to their unique anatomy and function, astrocytes sense and integrate multiple metabolic signals, including peripheral endocrine hormones and nutrients. Astrocytes and synapses engage in a complex dialogue of energetic and immunological interactions. Astrocytes act as a bridge between MetS and cognitive dysfunction, undergoing diverse activation in response to metabolic dysfunction. This article summarizes the alterations in astrocyte phenotypic characteristics across multiple pathological factors in MetS. It also discusses the clinical value of astrocytes as a critical pathologic diagnostic marker and potential therapeutic target for MetS-associated cognitive dysfunction.

## 1 Introduction

MetS is a syndrome of multiple metabolic disorders that seriously jeopardize cardiovascular health. MetS leads to myocardial metabolic, hemodynamic, and microcirculatory dysfunction by activating the sympathetic nervous system, the renin-angiotensin system, and pro-inflammatory adipokines (1, 2). Combining several recognized diagnostic criteria, the main diagnostic features of MetS include abdominal obesity, dyslipidemia, hyperglycemia, insulin resistance (IR), and elevated blood pressure (3–10). The multivessel risk factors of MetS jeopardize the cerebral vasculature and reduce cerebral perfusion while accelerating neuronal cell senescence and degeneration (11, 12). Evidence from diverse studies supports the association of MetS with vascular dementia and Alzheimer’s disease dementia (13). Various components of MetS have been found in cross-sectional and longitudinal studies to cause decreases in learning memory, attention, visuospatial and executive functions, and processing speed (14, 15). A 15-year follow-up analysis of 176,000 non-demented participants found that MetS led to a 12% increased risk of developing all-cause dementia (16).

Astrocytes are the primary glial cells and are essential for maintaining brain homeostasis. Astrocytes endfeet envelop neurons and the cerebral vasculature, linking cerebrovascular nutrient uptake transport to high oxygen- and sugar-dependent synaptic activity. Astrocytes shape synapses and their surrounding microenvironment, regulate cerebrovascular structure and perfusion, and influence neuroinflammation. Astrocytes become “reactive astrocytes” when stimulated by metabolic changes (e.g., glucose and lipid metabolism) (17). Reactive astrocytes are traditionally thought to have a double-edged role in cytotoxicity and neuroprotection (18). Cytotoxicity of astrocytes is defined as driving pathologic progression through the release of toxic factors such as inflammatory cytokines. Neuroprotective effects are usually heavy in ischemic injury, and reactive astrocytes promote vascular repair and remodeling. Escartin et al. (19) have pointed out the shortcomings of this binary division in recent years based on transcriptomic studies, suggesting that heterogeneity of reactive astrocytes should be emphasized.

Chronic low-level inflammatory states, peripherally and centrally, and systemic IR, are critical in the MetS, leading to cognitive dysfunction (20). A more detailed understanding of the underlying molecular mechanisms of MetS-related cognitive dysfunction will facilitate the development of new approaches to prevention and treatment. MetS-related nutritional and hormonal changes can significantly alter blood metabolic signaling, thereby regulating astrocytes’ responsive activation and specific genomic programs and functional transitions (17). Astrocyte activation is often considered an adaptive mechanism for metabolic adaptation and relief of neuronal stress, but it can also have multifaceted effects on cognitive function and metabolic homeostasis (21). However, persistent astrocyte proliferation and neurotoxic phenotype are essential causes of neuroinflammatory spread and chronicity (18, 22). Identifying the activation state of these astrocytes and the associated molecular mechanisms may provide new targets for treating MetS-related cognitive dysfunction (23).

In this review, we overview the multifaceted role of astrocytes in MetS-related cognitive impairment. Recent discoveries on astrocyte subpopulations and their regulation of cognitive and metabolic functions are highlighted.

## 2 MetS and cognitive dysfunction

**Figure 1:**
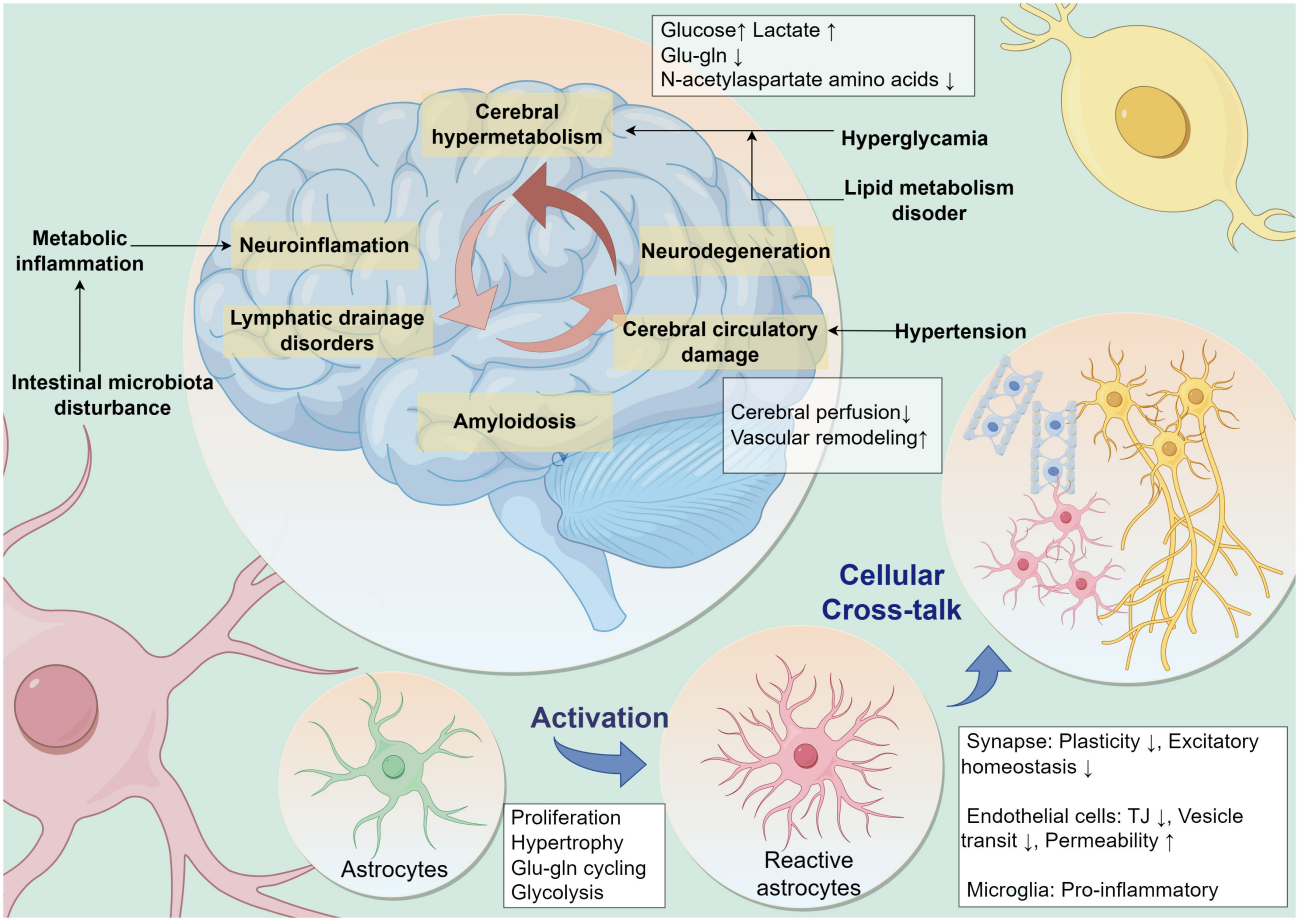
MetS leads to cognitive dysfunction through multiple pathologic factors. Multiple peripheral metabolic disorders in MetS, such as persistent hyperglycemic states, disorders of lipid metabolism, hypertension, metabolic inflammation, and disturbances in intestinal microbiota, are contributing to neurocognitive decline. Neuropathology in the brain leads to a vicious cycle of cognitive decline caused by neuroinflammation, cerebral microcirculatory dysfunction, impaired glial lymphatic system drainage, and accumulation of pathologic proteins. Astrocytes are potentially central to this vicious cycle. Metabolic stress pressure drives reactive activation of astrocytes and influences their interactive dialog with surrounding cells. Glu-gln, glutamate-glutamine; TJ, Tight Junction.

Neuroimaging changes associated with MetS have been observed in clinical studies, including reduced gray matter volume, cerebral white matter microstructural changes, cerebral atrophy, and lacunar cerebral infarcts (24, 25). Reduced resting-state functional connectivity between MetS-related vascular risk factors and multiple higher-order cognitive function-related neural networks (26). MetS leads to cerebrovascular injury and neurodegenerative lesions through complex mechanisms that ultimately produce altered cognitive function (27) (**Figure 1**).

MetS components such as low-density lipoprotein (LDL), high-density lipoprotein (HDL), hypertension, and advanced glycation end products (AGEs) accumulation all contribute to cerebral atherosclerosis, accelerating white matter damage, lacunar microinfarcts, and microhemorrhages (28, 29). The cerebral circulatory system has the capacity for adaptive regulation to maintain cerebral perfusion in fluctuating arterial blood pressure. MetS contributes to decreased cerebral microvascular density and blood flow, impairing cerebrovascular responsive autoregulation and blood flow reserve functions (30, 31). Excessive or unstable arterial blood pressure levels reduce cerebrovascular autoregulation, leading to cerebral hypoperfusion and neurodegenerative pathologies (32) and amyloid-β (Aβ) protein deposition by mechanical stretch (33). Stimulation by high blood pressure arterial wall shear stress increases vascular smooth muscle cell hypertrophy and proliferation, leading to vascular remodeling (34, 35). High-fat diet(HFD) rats were more sensitive to ischemia-reperfusion injury. Compared to normal diet rats, they exhibited more significant decreases in cerebral blood flow (CBF) and elevated levels of oxidative stress (36).

Oxidative stress and mitochondrial malfunction in MetS also promote neuroinflammation and neurodegeneration (37). High levels of circulating inflammatory markers are a common feature of MetS and cognitive dysfunction (38, 39). Ectopic fat accumulation, fatty inflammation (40), and disruption of intestinal flora (41) during the MetS disease process lead to a chronic low-grade inflammatory state. High levels of circulating pro-inflammatory cytokines in MetS disrupt and cross the blood-brain barrier (BBB) into the brain, thereby activating astrocytes and microglia to trigger a neuroinflammatory response, leading to a critical mechanism of MetS-related cognitive dysfunction (42, 43). Peripheral inflammation leads to neuroinflammation and oxidative stress, impairing energy supply to synaptic mitochondria (44).

## 3 Neurocognitive perspectives of astrocytes

Astrocytes serve as a communication bridge between the central and peripheral nervous systems. They are essential components of the neurovascular unit and communicate extensively with neurons, endothelial cells, and glia via dendritic structures (45). The complex and diverse dynamic network of astrocytes is the anatomical basis for their maintenance of metabolic and immune homeostasis of the brain. The astrocyte network integrates nutrient metabolic signals and interacts with hypothalamic functional neurons to exert central feedback regulation of appetite regulation, glucose sensing, and other systemic metabolism (46–48).

### 3.1 Astrocyte participate in neurosynaptic activity

Synaptic plasticity and presynaptic vesicle release in neurons are the mechanisms underlying working memory. Astrocytes are a new target for improving cognitive function, forming glial isolates that wrap 50-60% of brain synapses and regulate synaptic plasticity through glycolytic energy supply and release of multiple neuroactive substances (e.g., glutamate, ATP, adenosine, and D-serine) (49). Astrocytes regulate the neurosynaptic microenvironment with their unique glial-isolated structure and immunometabolic properties (50, 51). Perisynaptic astrocyte processes (PAPs) constitute the glial segregation of the synaptic gap. Astrocytes regulate glutamate concentration through glutamate transporter subtype 1(GLT-1) and deliver lactate through monocarboxylate transporters1 and 4 (MCT1, MCT4). Aging and atrophy of PAPs are accompanied by a decline in glutamate clearance and a decrease in Ca^2+^ events, and excess glutamic acid spillage in the synaptic gap will activate N-methyl-D-aspartic acid (NMDA) receptors to reduce the amplitude of the neuron’s long-term potentiation (LTP) (52). Furthermore, astrocytes control synaptic plasticity by producing neurotrophic factors, releasing and clearing neurotransmitters, and regulating ion levels in the extracellular environment (53).

In addition to regulating synapses, astrocytes regulate the electrical activity of neuronal networks by releasing gliotransmitters such as ATP, glutamate, and Ca^2+^ wave oscillations (54). Computational model studies of working memory suggest that astrocytes can store traces of neuronal activation in information processing (55). In contrast, memory extraction depends on astrocytes’ modulation of spiking neuron network connections (56). Astrocytes influence memory performance through states of conscious vigilance and basal arousal. Sustained neuronal firing in the hippocampus induces astrocytic γ-aminobutyric acid G protein-coupled receptor signals that control the oscillatory activity of the θ and γ oscillations of the hippocampal neuronal network (57).

Astrocytes coordinate interneuronal network projections between the hippocampus and cortex, thus participating in memory consolidation and storage. Astrocytes modulate communication between hippocampal CA1 and cingulate cortex to promote memory consolidation and retention (58). Dysfunctional cell division in astrocytes impairs hippocampal-prefrontal theta synchronization (59).

### 3.2 Astrocyte maintain brain energy metabolism

The neural axons and telopods of astrocytes wrap around the cerebral vascular system to detect nutrients and metabolic hormones in the arterial blood that enters the brain (60). Astrocytes form an intercellular communication network with each other through connexins, bridging the intravascular nutrient supply with the energy demand of neuronal activity. The astrocyte network enables the propagation and sharing of small molecule nutrient metabolic signals at the cellular network level (61). Astrocytes are the primary cells for glycogen storage and glycolysis in the brain. They shape the fundamental pattern of brain energy metabolism by coupling with neuronal oxidative phosphorylation (62, 63). Astrocytes can bridge the transient energy demands of neurosynaptic activity with the supply of circulating fuel (64).

Astrocytes store lipid droplets (LDs) by taking up excess fatty acids (FAs) from neurons via rich acid transport protein and lipid transport protein, making them critical sites for fatty acid oxidation. Astrocytes take up FAs produced by neuronal metabolism and help highly active neurons relieve lipid oxidative stress through astrocyte consumption of LDs for ATP production via NMDA receptor-mediated mitochondrial β-oxidation (65).

Recent studies have shown that astrocyte function is equally dependent on mitochondria and oxidative phosphorylation (66). Mitochondrial disorders in astrocytes affect brain oxidative phosphorylation metabolism and contribute to forming metabolic stresses such as reactive oxygen species (ROS) (67). Astrocytes can exert neuroprotective effects by delivering mitochondria to neurons. Peroxisome proliferator-activated receptor gamma coactivator-1 alpha (PGC-1α) and mGluR5(metabotropic glutamate receptor 5) modulate the mitochondrial network of astrocyte cells and produce positive Ca^2+^ signaling ion conductance to synapses (68). Restoring mitochondrial biogenesis in astrocytes may be a therapeutic target for neuropsychiatric disorders with impaired synapse formation.

### 3.3 Astrocyte control of the cerebrovasculature

The endfoot of astrocytes envelop the endothelium of the cerebral microcirculation. Astrocytes rapidly regulate CBF to meet neurocognitive energy demands, ensuring nutrient and oxygen delivery (69). Arachidonic acid, prostaglandin E2, and nitric oxide produced by astrocytes connect directly with small smooth muscle cells in the arteries (70). Additionally, astrocytes monitor cerebral perfusion pressure and regulate blood flow by modulating the diameter of cerebral arteries through their unique anatomical location and pressure-sensitive membrane structures (71). The end-foot of astrocytes abutting endothelial cells expresses the transient potential receptor vanilloid 4 (TRPV4) and is influenced by transmural pressure in penetrating arterioles and blood flow levels (72).

The endfoot of astrocytes with tight junction (TJ) proteins are the basic structures that comprise the BBB (73). Ablating astrocytes increases BBB permeability and impairs repair (74). Astrocytes regulate the TJ structure through the vascular permeability factor, matrix metalloproteinase (75). Astrocyte endfoot around blood vessels are tightly connected by connexins 30 (Cx30) and connexins 43 (Cx43), which allow ion exchange in the peri-endothelial astrocyte network. Deletion of these connexins weakens the BBB, leading to its opening under increased hydrostatic vascular pressure (76). Physiologically, astrocytes Cx30 and Cx43 are involved in memory formation (77). Astrocyte Cx43 controls the rate of synaptic vesicle release to regulate presynaptic function, controls glutamate levels and allows glutamine release to maintain synaptic transmission (78). Astrocyte-specific Cx30 and Cx43 double knockouts lead to widespread activation of astrocytes and microglia, significant suppression of neuronal excitability, excitatory synaptic transmission in hippocampal CA1 region, and decrease of spatial learning and memory (79). In addition, astrocytes secrete a variety of vasoactive substances, among which angiopoietin-1 (ANG-1), sonic hedgehog (SHH), and insulin-like growth factor-1 (IGF-1) protect the BBB. In contrast, vascular endothelial growth factors (VEGF), matrix metalloproteinases (MMP), nitric oxide, glutamate, and endothelin-1 lead to structural damage of the BBB (80). Damage to astrocyte structures reduces BBB permeability, thus allowing pathogens and toxins to enter the central nervous system (81). Peripheral inflammatory factor across BBB and drives the pro-inflammatory phenotype of glial cells. This is a crucial pathway by which systemic inflammation triggers neuroinflammation (82).

## 4 MetS alters astrocyte phenotype

Astrocytes undergo adaptive activation both anatomically morphologically and functionally as they respond to and maintain homeostasis in the brain’s internal environment. Both clinical and animal studies have found that disorders of glucolipid metabolism and hypertension can induce reactive activation of brain astrocytes and overexpression of glial fibrillary acidic protein (GFAP) (83). Astrocytes, as reactive cells, are regulated by complex factors of circulating origin. Different types of stimuli induce specific reactive changes. In addition, sex was an essential variable in the analysis of MetS and cognitive dysfunction, and there was sex- and age-related heterogeneity in the altered responsiveness of astrocytes (84). The prevalence of MetS and related complications is higher in men than in women at ages younger than 50 years. The risk of MetS and associated complications in women exceeds that of men, with a decline in estrogen levels after menopause (85, 86). Recent reports suggest that astrocyte numbers, differentiation, and function differ between the sexes. Sex differences in reactive astrocytes are responsible for the emergence of sex differences in neuroendocrine regulation and cognitive function. Astrocytes isolated from female rats were more resistant to cell death induced by hypoxia, palmitic acid (PA), and lipopolysaccharide (LPS) than male astrocytes (87–89).

### 4.1 Hypertension

Increased numbers and morphological hypertrophy of reactive astrocytes in the brain have been observed in several animal models of hypertension (**Table 1**). 26-week-old SHR showed increased numbers and areas of immunoreactive positive astrocytes in the prefrontal cortex, occipital cortex and significantly higher numbers of GFAP immunoreactive positive astrocytes in the hippocampal region, compared to the same-week-old WKY rats (94). In the chronic hypertension model induced by 8 weeks of AngII infusion, a linear positive correlation between astrocyte morphology and elevated arterial blood pressure proliferated in cerebral white matter (95). Astrocyte endfoot are in contact with cerebral blood vessels, directly sensing circulating hemodynamic changes and releasing vasoactive substances to modulate slight arterial tone to maintain CBF independent of blood pressure fluctuations (96). Astrocyte reactive activation accompanied by transient potential receptor vanilloid 4 (TRPV4) activation was observed in the hippocampus of an AngII 28-day injection-induced mouse model of chronic hypertension. In this study, astrocyte TRPV4 mediated an increase in spontaneous Ca^2+^ events within microdomains, which enhanced parenchymal arteriole tone and decreased cognitive function (90).

**Table 1:** Effects of hypertension on astrocyte pathology and cognitive functions.

Experimental animal			Brain region	Astrocyte Phenotypes		Potential/associated impacts			Molecular mechanisms	Reference
Animal	Model	Control		Activation	Dysfunctions	Neuropathology		Behavioral		
C57BL6 (male), 8 weeks/NM	Ang II for 14 or 28 days (pump in,600 ng/kg/min)	NM	Cortex	GFAP↑ Number of cells↑	Ca2+ activity↑	PA tone↑ Myogenic responses↑		NM	TRPV4 channel	Ramiro et al., 2019 (90)
SD rats(male), 8 weeks/200-230 g	Partial occlusion of left renal artery	Renal artery was only exposed but not occluded	Cortex Hippocampus	GFAP↑	TRAF6↑ IκB-α↓ pP38↓ pERK1/2↓	NM		NM	CD40L	Ali et al., 2017 (91)
SHRs (male) 32 and 64 weeks/NM		WKY rats (male) 32 and 64 weeks/NM	Hippocampus	Cell body↑ Branches↑	PPARγ↓	Bax↑ Bcl-2↑ Caspase-3↑ INOS↓ Gp47phox↓		NM	NM	Yali et al., 2016 (92)
SD rats(male), 8 weeks/NM	Partial occlusion of left renal artery	Renal artery exposed	Cortex Hippocampus	GFAP↑ Processes↑ Cell body↑	NM	NM		NM	NM	Shahnawaz Ali et al., 2018 (93)

Bax, Bcl-2-associated X protein; Bcl-2, B-cell lymphoma 2; CD40L, CD40 Ligand; GFAP, Glial fibrillary acidic protein; pERK, Phospho extracellular regulated protein kinases; SD rat, Sprague dawley rat; SHR, Spontaneously hypertensive rats; TRAF6, TNF receptor associated factor; TRPV4, Transient receptor potential vanilloid 4; PPARγ, Peroxisome proliferator-activated receptor γ; iNOS, Inducible nitric oxide synthase; NM, Not mentioned. ↑, Increase; ↓, Decrease.

Studies in hypertensive humans and hypertensive rat models have shown that an overactive brain renin-angiotensin system (RAS), which leads to oxidative stress and neuroinflammation in several brain regions, including the brainstem cardiovascular centers and the hippocampus (97, 98). The overactive brain RAS in hypertension also contributes to cognitive dysfunction and exacerbates hypertension through sympathetic excitation. In several experimental and genetic models of hypertension, including spontaneously hypertensive rats (SHR) (99, 100) and desoxycorticosterone acetate salt hypertensive rats (101), hyperactivity of the central RAS was observed, especially increased levels of angiotensin II (AngII), angiotensin III (AngIII) and angiotensin II receptor type 1 (AT1R). *In vitro* studies have shown the expression of AT1R and AT2R on human astrocytoma cell lines (102)and primary cerebral cortex astrocytes (103). Therefore, it has been suggested that astrocytes may play a role in neuroinflammation and oxidative stress caused by AngII and AngIII in the brain RAS (102). Studies conducted on primary astrocytes isolated from SHR have shown that AngII causes the secretion of IL-6 from astrocytes through the activation of NF-κB/ROS and overexpressing cyclooxygenase 2 via astrocyte AT1R (104, 105). In primary rat astrocytes derived from SD rats, AngIII targets AT1R to activate extracellular regulated protein kinases (ERK)1/2 MAP kinases and c-Jun N-terminal kinase (JNK) phosphorylation to promote astrocyte proliferation (106).

### 4.2 Lipid metabolism disorders

Astrocyte activation and proliferation in the hippocampus and hypothalamus have been observed in HFD-induced obese rat models (**Table 2**) (114–117).

**Table 2:** Effects of hyperlipidemia and obesity on astrocyte pathology and cognitive functions.

Experimental animal			Brain region	Astrocyte Phenotypes		Potential/associated impacts			Molecular mechanisms	Reference
Animal	Model	Control		Activation	Dysfunctions	Neuropathology	Behavioral			
C57BL/6N mice (male),8 weeks/NM	HFD for 12 weeks	CD for 12 weeks	VAc	NM	GLAST↓ GLT-1↓	Glutamatergic inputs↑	Depression (SPT, FST)		NM	Tsai et al., 2022 (107)
C57BL/6 mice (male),7-8 weeks/20 g	HFD for 1 month	CD for 1 month	Hippocampus	GFAP↑	NM	BDNF↓ NLRP3↑ ASC↑ IL-1β↑ TNF-α↑	Depression and anxiety (OFT, EPM, SPT, FST)		NM	Li et al., 2022 (108)
C57BL/6 mice(male), NM/NM	HFD for 8 weeks + CSDS	CD for 8 weeks + CSDS	mPFC	Spreading area↑	D-serine↑, Glutamate↑	sIPSCs↓ sEPSCs↓	Depression (SPT, TST)		JNK–STAT3	Yu et al., 2022 (109)
C57BL/6J mice(male),6 weeks,NM	HFD for 12 weeks	LFD for 12 weeks		GFAP↑	AQP4↓	GS functions↓ CBF↓ Neuropathological alterations	Cognitive dysfunction (MWM)		NM	Zhan et al., 2024 (110)
Long-Evans rats (male), NM/NM	Cafeteria diet for 40 days	CD for 40 days	OFC	GFAP↑ Astrocyte hypertrophy↑	GLT-1 function↓	LTD of GABA transmission↓	NM		NM	Lau et al., 2021 (111)
SD rats (male), NM/250-270g	HFFD for 7 days	CD for 7 days	Hippocampus	GFAP+ cell number↑ GFAP area↑	NM	NM	NM		NM	Erika et al, 2014 (112)
WKY rat(male),6 weeks/NM	HFrD for 12 weeks	CD for 12 weeks	Hippocampus	GFAP↑	NM	NM	NM		NM	Liu et al., 2018 (113)

AQP4, Aquaporin 4; BDNF, Brain-derived neurotrophic factor; CBF, Cerebral blood flow; CD, Control diet; CSDS, Chronic social defeat stress; EPM, Elevated plus maze; FST, Forced swim test; GFAP, Glial fibrillary acidic protein; GLAST, Glutamate aspartate transporter; GLT-1, Glutamate transporter-1; GS, Glutamine synthetase; HFD, High-fat diet; HFFD, High-fat and High-fructose diet; HFrD, High-fructose diet; IL-1β, Interleukin-1 beta; JNK, c-Jun N-terminal kinase; STAT3, Signal transducer and activator of transcription 3; LTD, Long-term depression; LFD, Low-fat diet; MWM, Morris water maze; MyD88, Myeloid differentiation primary response 88; NLRP3, NOD-like receptor thermal protein domain associated protein; NM, Not mentioned; OFT, Open field test; POMC, Pro-opiomelanocortin; SPT, Sucrose preference test; TST, Tail suspension test; TNF-α, Tumor necrosis factor alpha; VAc, Ventral hippocampus; sEPSCs, Spontaneous excitatory postsynaptic currents; sIPSCs, Spontaneous inhibitory postsynaptic currents. ↑, Increase; ↓, Decrease.

HFD-induced obesity also affects astrocyte lipid oxidation and mitochondrial metabolism. Fatty acid β-oxidation (FAO) in the brain occurs mainly in astrocytes. Astrocytes store overloaded free fatty acids as LDs, which are used via FAO to energize neurons and protect them from lipotoxic damage. Obesity leads to the accumulation of astrocyte misfolded proteins, induced endoplasmic reticulum stress, and thus crosstalk with neurodegenerative (21). Obesity decreases fatty acid oxidation in hypothalamic astrocytes, leading to disturbed mitochondrial dynamics (118). In the hippocampus of mice raised on a HFD for 1 month, astrocytes’ lipid and cholesterol content was elevated, accompanied by an increase in the number of secondary branches and lobules of neural protrusions (119). *In vitro* studies have found that saturated and unsaturated fatty acids have opposite regulatory effects on astrocyte lipoprotein lipase (LPL). TGs and palmitic acid decrease LPL expression and oleic acid elevate LPL. HFD-induced elevation of LPL in hypothalamic astrocytes of obese rats increases the accumulation of LDs. It impairs glycolytic metabolism, impairing glucose tolerance, increases food intake, and aggravates obesity (120).

In addition, obesity and pathologic fat accumulation lead to decreased function of astrocyte-neuron crosstalk, in which high serum levels of leptin inhibit astrocyte excitatory amino acid transporter protein (EAAT) expression and promote sympathetic overactivation (121). Obesity impairs glutamate clearance from the synaptic gap by astrocytes and attenuates the endogenous cannabinoid pathway and the synaptic plasticity it mediates in vertebral neurons in the orbitofrontal cortex (111). Astrocytes present a compensatory neuroprotective effect in the early stages of lipid metabolism disorders and are progressively bettered by chronic stressful pressures. It was found that 8 weeks of HFD induced astrocyte proliferation and limited neuronal damage by releasing heat shock protein 70 (HSP70) and ciliary neurotrophic factor (CNTF). In contrast, the compensatory neuroprotective effect of astrocytes was depleted after 20 consecutive weeks of HFD (122).

### 4.3 Glycemic derangement

High blood glucose levels in both *in vivo* and *ex vivo* studies resulted in reactive activation of astrocytes accompanied by changes in metabolic processes (**Table 3**). The astrocyte glycolytic effect is two-sided, with increased glycolytic flux supplying neurons with energy and antioxidants (133), while glycolysis also provides energetic support for inflammatory responses (134). A comprehensive review of astrocyte glycolysis in cellular metabolic immunity is lacking, but some studies have suggested that it undergoes the same progression from compensation to decompensation as immune cells (135). Multiple *in vitro* studies have found increased glycogen content and glycolytic activity in astrocytes chronically exposed to high glucose (136). In a ^1^H NMR-based metabonomic approach study, an increase in glucose uptake, glycolytic activity lactate release, and downregulation of TCA cycling activity were found in astrocytes after 72 hours of high-glucose exposure (137). High glucose promotes glucose uptake and glycogen storage in primary astrocytes but reduces maximal respiratory and glycolytic reserve capacity (138). It is suggested that high glucose leads to an increase in astrocyte glucose metabolic flux, but the efficiency of cellular energy utilization is reduced, making it more vulnerable to stressful pressures.

**Table 3:** Effects of hyperglycemia on astrocyte pathology and cognitive functions.

Experimental animal			Brain region	Astrocyte Phenotypes		Potential/associated impacts			Molecular mechanisms	Reference
Animal	Model	Control		Activation	Dysfunctions	Neuropathology	Behavioral			
C57BL/6J mice(male), 8 weeks/NM	HFFD for 9 weeks	CD for 9 weeks	Hypothalamic	GFAP↑ Vimentin ↑	HMG20A↑	NM	NM		HMG20A	Petra I et al., 2021 (123)
C57BL/6N mice(male), 8 weeks/NM	HFD for 4 weeks (from 20 to 24 weeks old)	CD for 4 weeks	Ventral hippocampal	Process lengths, branch points, intersections↓ GFAP↑	NM	NM	Depression (OFT, EPM) Cognitive dysfunction (ORT)-		NM	Ying-Yiu et al., 2021 (124)
Wistar rats(male), 6 weeks/NM	HFD for 50 days + STZ (35mg/kg bw i.p)	CD for 50 days+ sodium citrate buffer i.p	Hippocampus	GFAP↑	NM	NM	NM		NM	Velia et al., 2022 (125)
C57Bl/6 J mice(male), 4 weeks/NM	HFD for 17 weeks	CD for 17 weeks	Hippocampus CA1 DG	GFAP↑	NM	NM	NM		NM	Saieva et al., 2022 (126)
			POCTX	GFAP↑						
			FCTX	GFAP-						
SD rats(male), 8 weeks/200-230 g	HFD for 16 weeks + STZ (40mg/kg on 5 consecutive days i.p)	CD for 16 weeks	ARC of hypothalamus	GFAP↑	PDK2 p-PDH↑	Tnf-α↑ Il-1β↑ Il-6↑ NPY/AgRP neurons↑	Feeding behavior dysregulation		PDK2-lactic acid axis	Rahman et al., 2020 (127)
C57BL/6 mice(male), 6 weeks/NM	MLDS STZ (40mg/kg on 5 consecutive days i.p)	Sodium citrate buffer i.p	Hippocampus	GFAP↑ Hypertrophic morphology	NM	Tnf-α↑ Il-6↑	Cognitive dysfunction (NOR, Y maze)		LCN2 ↑	Anup et al., 2019 (128)
Obese Zucker rats(male), 12 weeks/NM		LZRs, 12 weeks/NM	Hippocampus	GFAP↑	NM	NM	NM		NM	Daniele et al., 2013 (129)
KK-Ay mice(male), 5 months/NM +HFD for 3 months		C57BL/6J mice male), 5 months/NM + CD for 3 months	Hippocampal	Cell body↓ Branches↓	vGLUT1↑ GLUT1↓ EAAT2-GDNF↓	IL‐1β↑ TNF‐α↑ BDNF↓	Cognitive dysfunction (MWM)		NM	Si et al., 2020 (130)
db/db(male), 15 weeks/NM		C57BLKS/J(male), 15 weeks/NM	Hippocampal	GFAP↑	Glu-gln cycle↑ GAD↑ GLS↑ GS↑ Lactate↑ Taurine↑ Pyruvate↓ Succinate↓ Citrate↓	TUNEL↑	Cognitive dysfunction (MWM)		NM	Yongquan et al., 2016 (131)
db/db(male), 8 weeks/NM		C57BLKS/J(male), 8 weeks/NM	Hippocampal	GFAP↑	C3↑ S100A10↓	IL-6↑ IL-1β↑ TNF-α↑ IL-18↑ TfR1↑ DMT1↑ FPN1↓ MDA↑ SOD↓ GSH↓ ROS↑	Cognitive dysfunction (MWM)		NM	Ji-Ren et al., 2023 (132)

AgRP, Agouti-related peptide; ARC, Arcuate nucleus; BDNF, Brain-derived neurotrophic factor; CD, Control diet; C3, Complement component 3; DMT1, Divalent metal transporter 1; db/db, Diabetic (leptin receptor deficient) mice; EPM, Elevated plus maze; EAAT2, Excitatory amino acid transporter 2; FCTX, Frontal cortex; FPN1, Ferroportin 1; GAD, Glutamate decarboxylase; GDNF, Glial cell-derived Neurotrophic Factor; GFAP, Glial fibrillary acidic protein; GLS, Glutaminase; GLUT1, Glucose transporter 1; GSH, Glutathione; HFD, High-fat diet; HFFD, High-fat and high-fructose diet; HMG20A, High Mobility Group 20A; Il-1β, Interleukin 1 Beta; IL-6, Interleukin 6; IL-18, Interleukin 18; LCN2, Lipocalin-2; MDA, Malondialdehyde; MWM, Morris water maze; NPY, Neuropeptide Y; NM, Not mentioned; NOR, Novel object recognition test; OFT, Open field test; ORT, Object recognition test; PDK2, Pyruvate dehydrogenase kinase 2; POCTX, Posterior cortex; p-PDH, Phosphorylated pyruvate dehydrogenase; ROS, Reactive oxygen species; S100A, S100 protein; SOD, Superoxide dismutase; STZ, Streptozotocin; TfR1, Transferrin receptor 1; Tnf-α, Tumor necrosis factor alpha; TUNEL, Terminal deoxynucleotidyl transferase dUTP nick end labeling; vGLUT1, Vesicular glutamate transporter 1; Y maze, Y-shaped maze task. ↑, Increase; ↓, Decrease.

Astrocyte metabolic plasticity has a double-edged role, feeding the inflammatory immune response process and acting as a buffer against metabolic stress. The astrocyte pentose phosphate pathway and glutathione levels increase with blood glucose, reduce ROS production, and protect neurons from oxidative stress damage (139). *In vitro* metabolomics studies have found that astrocytes produce and transport more lactate in high-sugar environments, which may work to enhance astrocyte-neuron lactate shuttling (137).

High glucose leads to increased secretion of multiple pro-inflammatory factors by astrocytes, leading to neuroinflammation (140). High glucose increases the expression and secretion of pro-inflammatory cytokines IL-6 and IL-8 in human primary astrocytes and U-118MG astrocytoma cells via STAT-3 (141). Hyperglycemia induces enlargement of astrocytes in the hippocampus and is linked to peripheral recruitment of leukocytes to the cerebrovascular system (142). High glucose exacerbates neuroinflammation via ROS/mitogen-activated protein kinase (MAPK)/NF-κB, ERK, and JNK pathways by upregulating matrix metalloproteinase-9 expression in rat brain astrocytes (143, 144). The toll-like receptor (TLR) of astrocytes serves as an essential target of the innate immune system, and high glucose promotes neuroinflammation and altered cellular metabolism via the TLR/MAPK/PPARs pathway (145).

Astrocyte responsiveness to high glucose affects microcirculatory endothelial barrier structure. Hyperglycemia induces increased secretion of VEGF protein in astrocytes, impairment of gap junctional Cx43 and Cx30 proteins, and reduced transendothelial cell electrical resistance (TEER), which are critical factors for reduced BBB permeability (146, 147). Sustained hyperglycemia induces the non-enzymatic glycosylation of various proteins and the resulting formation of advanced glycation endproducts (AGEs), which mediate the development of diabetic complications by targeting the receptor of advanced glycation endproducts (RAGE). Primary astrocytes from mice cultured with high glucose showed increased expression of immune complement C3 and decreased synaptic number, suggesting that high glucose promotes synaptic phagocytosis of the complement pathway in astrocytes. In this study, the RAGE-p38MAPK-NF-κB pathway was a vital upstream of the synaptic phagocytosis promoted by high glucose in astrocytes (148).

## 5 MetS leading to cognitivedysfunction via astrocyte pathology

**Figure 2:**
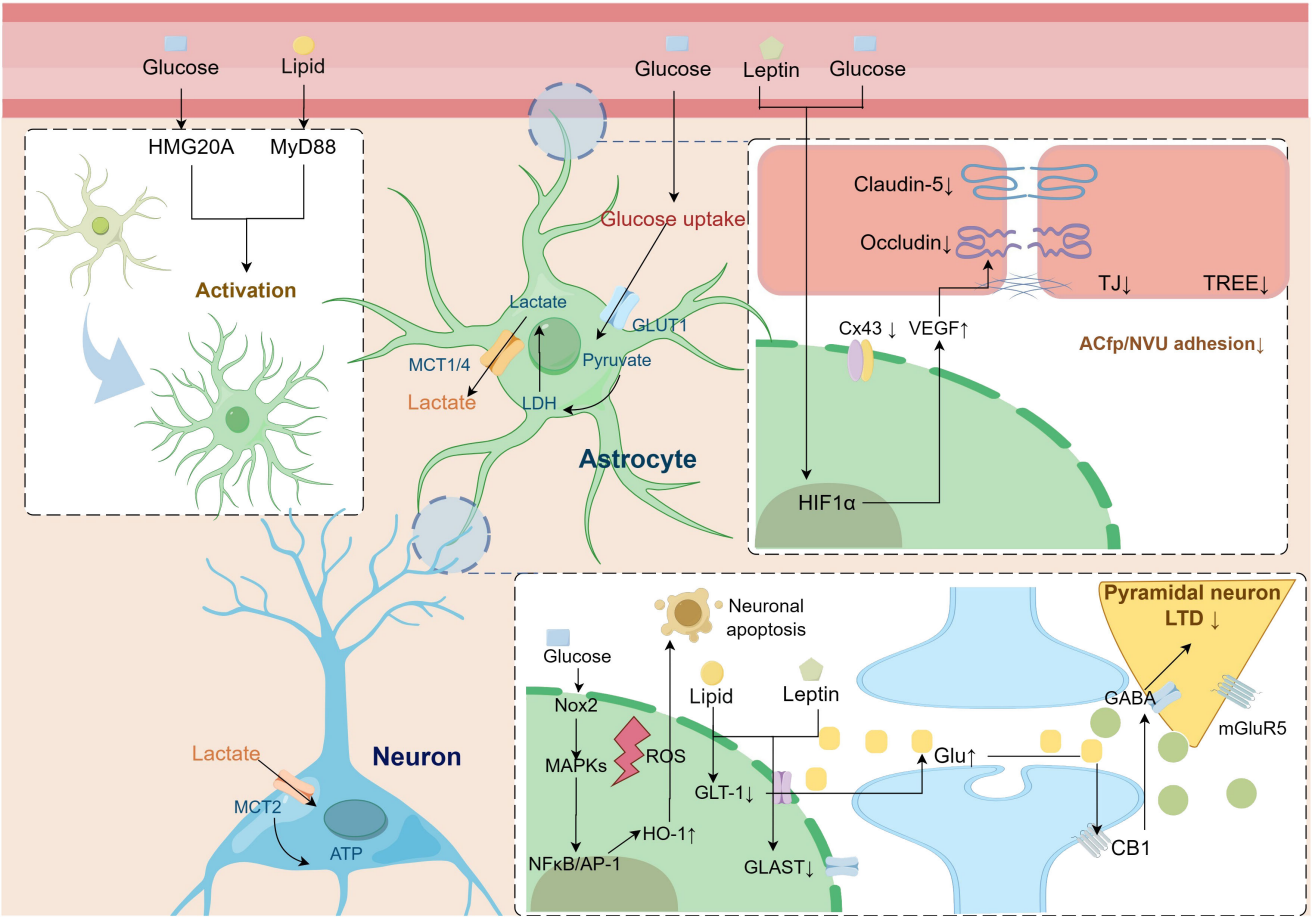
Pathologic alterations of astrocytes in metabolic syndrome leading to cognitive dysfunction. Astrocytes undergo reactive activation driven by a disturbed metabolic state. Activation of the astrocyte HIF-1α pathway secretes multiple factors, including VEGF, that impair BBB-linked proteins, leading to increased blood-brain barrier permeability. At the same time, the lipid, glucose, and amino acid metabolism coupling pathway between astrocytes and neurons is impaired, leading to an imbalance in neuronal excitability and decreased synaptic plasticity. AP-1, activator protein-1; CB1, cannabinoid 1; Cx43, connexin 43; GABA, γ-aminobutyric acid; GLAST, glutamate aspartate transporter; Glu, glutamate; GLUT1, glucose transporter type 1; GLT-1, glutamate transporter subtype I; HIF1-α, hypoxia-inducible factor1-α; HMG20A, high mobility group domain protein 20A; HO-1, heme oxygenase-1; LDH, lactate dehydrogenase; LTD, long-term depression; MAPK, mitogen-activated protein kinase; MCT, monocarboxylate transporter; mGluR5, metabotropic glutamate receptor 5; MyD88, myeloid differentiation primary response 88; NFκB, nuclear Factor-κB; NOX, nicotinamide adenine dinucleotide phosphate oxidase; ROS, reactive oxygen species; TJ, tight Junction; TREE, trans-endothelial/epithelial electrical resistance; VEGF, vascular endothelial growth factor.

The hippocampus is a major brain region involved in memory functions, and its synaptic plasticity activities of vesicular and ionic channel activity depend on continuous energy support and are susceptible to nutrient metabolism (149). The release of gliotransmitters in astrocytes modulates neural theta oscillations between the dorsal hippocampus and prefrontal cortex, which are involved in memory formation and storage (59). Morphologic, immunologic, and metabolic alterations in astrocytes mediate the contribution of multiple factors to the development of cognitive dysfunction in MetS (**Figure 2**).

### 5.1 Astrocyte morphologic alteration

In MetS, astrocytes undergo reactive changes in cell morphology, as demonstrated by increased cell proliferation and hypertrophy, neural protrusion density and axon length. It was found that glial segregation and TJ barrier structures constituted by astrocytes proliferating their endfoot can limit neuroinflammatory damage (73). The morphologic plasticity of astrocytes is compatible with their functional transformation to limit the spread of inflammation and support nerve regeneration (150).

The altered morphology of astrocytes leads to impaired communication with other cells in the neural vascular unit, which in turn impairs the barrier immunity and energy substance uptake function of the BBB (151). Both *in vivo* and *ex vivo* studies have shown that a hyperglycemic state impairs gap junction communication in astrocytes, inducing swelling of the astrocyte endfoot and detachment from the endothelial cell basement membrane (146). Astrocyte endfoot processes (ACfp) from the neurovascular unit (NVU) were observed in the prefrontal cortex of female diabetic db/db mice. Loss of ACfp/NVU adhesion has been suggested as a potential mechanism contributing to impaired cognitive function in diabetes (152). Obesity induces reactive proliferation of astrocytes, which in turn induces structural remodeling of the neuroglial interface in multiple brain regions and alters the immune and transport functions of the BBB (153, 154). HFD-induced glial proliferation of astrocytes affects the BBB structure in the arcuate nucleus region. It makes it more difficult for neuropeptide Y (NPY) and proopiomelanocortin (POMC) neuronal cytosomes and dendrites in this region to reach the vasculature (155). Obesity is accompanied by increased serum leptin levels, which activate hypoxia-inducible factor 1-alpha (HIF-1α)-VEGF signaling in hypothalamic astrocytes, thereby inducing structural remodeling of the glial interface (156). Astrocytes isolated from stroke-prone spontaneously hypertensive rats (SHRSP) cause TJ damage and high resistance in endothelial cells by secreting large amounts of lactate (157).

Several studies have found that the overall neural processes of astrocytes are shortened in states of metabolic dysregulation, diminishing their modulation of synapses. A HFD for 12 weeks induced an increase in GFAP expression in the rat hippocampus but in turn impaired the length of neural protrusions in astrocytes, as well as the expression of the proteins glutamate aspartate transporter (GLAST), GLT-1, and Cx43, which are associated with synaptic plasticity (124, 158, 159). Chronic overnutrition leads to the shortening of the central neural protrusions of astrocytes through upregulation of the IκB kinase β (IKKβ)/nuclear factor-κB (NF-κB) pathway, which in turn affects their glutamate uptake in the synaptic gap and modulation of synaptic excitability (160).

### 5.2 Astrocyte immunoreactivity

Astrocytes are thought to be critical regulators of neuroinflammation (161). Peripheral immune signaling drives glial cell immune function switching as a potential mechanism for systemic inflammation to trigger neuroinflammation (82). Astrocytes play a vital role in developing and expanding neuroinflammation by interacting with various central nervous system *in situ* immune cells, including microglia and T cells (162, 163). Moreover, astrocytes drive perivascular leukocyte recruitment to the brain by secreting C-C motif chemokine ligand 2 (CCL2) and C-X-C motif chemokine ligand 10 (CXCL10) (163, 164). GFAP, S100 calcium-binding protein B (S100B), monoamine oxidase-B (MAO-B), chitinase-3-like protein 1 (YKL-40), and D-serine were used as biomarkers to assess the reactivity and proliferation intensity of astrocytes in tissues, cerebrospinal fluid, and blood (165, 166). High glucose activation of the complement 3 (C3) pathway in astrocytes can lead indirectly and directly to synaptic loss. C3 secreted by astrocytes is able to interact with microglial component 3a (C3a) receptors to modulate synaptic phagocytosis in microglia (167). Reduced secretion of complement factor C3/C3a in high glucose-treated primary astrocytes leads to synaptic protein damage and cognitive dysfunction (168).

As previously described, multiple factors in MetS, such as peripheral metabolic glucolipid metabolism disorders and metabolic inflammation, can drive astrocyte reactive activation. Several studies have demonstrated that enhanced GFAP immunoblotting is observed for T2DM disease states lasting 2-4 weeks, whereas GFAP expression is significantly reduced in more extended weekly studies (169, 170). HFD induces increased hippocampal GFAP expression in rats, which is associated with neuroinflammation, microvascular damage, and subsequent cognitive dysfunction (171, 172). The IKKβ/NF-κB of astrocytes is an essential pathway for HFD-induced hypothalamic inflammation. Knockdown of IKKβ in astrocytes can improve HFD-induced hypothalamic neuroinflammation, insulin resistance status, and glucolipid metabolism (173). HFD induces upregulation of hypothalamic potassium inwardly rectifying channel subfamily J member 2 Gene (*Kcnj2*), Complement 4b (*C4b*) and discoidin domain receptor 1 (*Ddr1*) and co-localizes with GFAP, and is therefore considered an early marker of obesity and diabetes-related cognitive dysfunction (174). Secretion of inflammatory factors by astrocytes is associated with synaptic loss. In neuron-astrocyte co-culture cell studies, LPS increased astrocyte secretion of inflammatory factors and correlated with decreased neuronal synaptophysin (SYN) (175).

MetS leads to the activation of astrocytes, thereby affecting their regulatory role in cognition and behavior. Reactive astrocytes are a potential therapeutic target for ameliorating vascular and neurodegeneration-related cognitive dysfunction (176, 177). Targeting the neurotoxic phenotype of reactive astrocytes alleviates cognitive-behavioral alterations induced by MetS-related factors (169).

### 5.3 Astrocyte immunometabolic disorders

Reactive astrocytes respond to metabolic stress by reprogramming metabolic processes and exerting various adaptive compensatory effects to maintain neuronal energy supply (178, 179). Various components of MetS can act directly on metabolic processes such as glycolysis and mitochondrial metabolism in astrocytes. Astrocyte metabolism progresses towards dysregulation under metabolic stress, with mitochondrial malfunction, energy failure, and oxidative stress, which can affect the energy supply of neurosynapses and impaired lymphatic efflux of Aβ proteins (180). Astrocyte glycolysis, gluconeogenesis, and lipid metabolism are plastic to undergo reprogramming during MetS metabolic stress to maintain neuronal energy homeostasis (181). Excessive chronic stressful pressure leads to a compensatory decrease in astrocyte energy metabolism, which may be impaired by promoting cerebral insulin resistance, decreased glucose uptake, and oxidative stress (66).

Central leptin signaling activation in HFD rats reduces astrocytic ghrelin transporter protein and EAAT1 and EAAT2 in the arcuate nucleus of the hypothalamus, resulting in decreased ghrelin uptake and reduced glutamine synthesis (121). Chronic lipid exposure-induced ectopic lipid loading in astrocytes leads to reduced insulin-induced protein kinase B (AKT) phosphorylation and dysregulated glycogen metabolism (182). The metabolomic study showed that selenium amino acid metabolism, urea cycle, and glutamate metabolism were up-regulated in human astrocytes in a palmitic acid-induced lipotoxic environment for several amino acid metabolic pathways (183). Several tricarboxylic acid cycle intermediates, such as succinate and citrate were reduced, glutamine synthetase was increased, and glutaminase and glutamic acid decarboxylase decreased in the hippocampal region of db/db mice (131). During physiological states, lipid synthesis and metabolism in astrocytes regulate hippocampal synapse development and function. Diminishing of sterol regulatory element-binding protein 2 (SREBP2) cleavage-activating protein (SCAP) resulted in lower levels of the synaptosome associated protein 25(SNAP-25) and reduced numbers of synaptic vesicles in the hippocampus of mice (184). Diabetes mellitus leads to impaired brain cholesterol synthesis and reduced synapse number by reducing the transcription factor SREBP2 (185).

Metabolic reprogramming of astrocytes is also thought to be an adaptive change in response to central insulin resistance (186). Astrocytes express insulin receptor and insulin-like growth factor 1 (IGF1), which regulate glucose transporter type 1 (GLUT1) expression to take up circulating glucose (187). In another study, IR knockdown in astrocytes was found to impair tyrosine phosphorylation of Munc18c, reduce ATP cytokinesis, and subsequently lead to reduced neuronal dopamine release and depressive-like behavior (188).

The process of reactive activation of astrocytes is accompanied by a metabolic paradigm shift (189). Activation and maintenance of reactive astrocytes depend on a continuous supply of energy from glycolytic metabolism. The downright inflammatory response of astrocytes to LPS is accompanied by elevated glycolytic flux and elevated activity of critical metabolic enzymes, such as 6-phosphofructose-2-kinase/fructose-2,6-bisphosphatase isoform 3 (PFKFB3) (190). 2-DG glycolysis inhibitor and glycogen phosphorylase inhibitor intervened to regulate the astrocyte glycolysis process and significantly attenuated LPS-induced cytokine release and NF-κB phosphorylation (134). Inhibition of pyruvate dehydrogenase kinase-2(PDK2) in hypothalamic astrocytes of diabetic rats inhibited cellular glycolysis and its inflammatory activation, thus reducing hypothalamic inflammation as well as lactate levels and reversing the increase in food intake (127). The peroxisome proliferator-activated receptor (PPAR) pathway is one of the critical pathways of astrocyte immunometabolism, among which PPARγ stimulates glucose and glutamate uptake and lactate release from astrocytes. At the same time, PPARα induces fatty acid β-oxidation in the presence of impaired glucose metabolism (191). Regulators of aerobic glycolysis, such as HIF-1α and AMPK in astrocytes, are affected by inflammation (192). Nicotinamide phosphoribosyltransferase (NAMPT)-dependent nicotinamide adenine dinucleotide (NAD^+^) upregulation in astrocytes provides energy for cellular activation and drives transcriptional inflammatory program rearrangements, and inhibition of endogenous NAD^+^ synthesis impairs astrocyte transcriptional responses to LPS/Interferon-γ(IFNγ) stimulation and attenuates activation-associated neuroinflammation (193).

## 6 Clinical practice of astrocyte in MetS-related cognitive dysfunction

In summary, it has been elaborated that astrocytes are susceptible to morphologically and functionally responsive changes in metabolically disturbed environments. Reactive astrocytes are involved in pathological processes such as neuroinflammation, energy metabolic homeostasis, and cross-barrier transport of nutrients and amyloid in MetS-associated cognitive dysfunction (165, 194).

### 6.1 Diagnostic markers

Astrocytes express and secrete a variety of specific molecular substances during pathological processes, which are considered promising targets for the development of early screening with humoral or imaging biomarkers (195). Several studies and meta-analyses have found a strong association between astrocyte biomarkers and cognitive decline (166). In a clinical study of 121 older adults, cerebrospinal fluid and plasma levels of GFAP and YKL-40 were shown to relate to Aβ and tau pathology and to mediate hippocampal volume atrophy (196).

Proteomic studies have identified increased levels of the metabolism-related proteins lactate dehydrogenase B-chain (LDHB), pyruvate kinase (PKM), and glyceraldehyde 3-phosphate dehydrogenase (GAPDH) in M4 Astrocyte in cerebrospinal fluid, which can be used as a biomarker for the early diagnosis of cognitive impairment (197). In addition, at the level of genetic material, astrocyte-derived extracellular vesicles (EVs) and a variety of miRNAs therein are thought to potentially become biomarkers for neurodegenerative diseases (198). Increased secretion of miR-141-3p and miR-30d is detected in primary human astrocytes activated by stimulation with the neuroinflammatory factor IL-1β (199). Regarding imaging, astrocyte metabolic levels and associated metabolites are visualized and analyzed by PET/CT imaging with 11C-acetate and 18F-fluorodeoxyglucose (18F-FDG). In a study, astrocyte acetate hypermetabolism and neuronal glucose hypometabolism were used as a visual diagnostic strategy for early diagnosis of Alzheimer’s disease (200). In addition, machine learning research is an emerging tool for constructing diagnostic models. In a study, automata theory was used to build a diagnostic computational model to monitor the astrocyte metabolic end-product lactate, thereby characterizing the level of glycogen metabolism in the brain (201).

Astrocytes have been progressively recognized as potential biomarkers for the development of cognitive impairment. At the same time, these markers are highly expressed in MetS and its related components. A joint examination of astrocytes in both diseases is still lacking. However, as reactive cells, their immunometabolic flexibility and sensitivity make them valuable as potential diagnostic markers of MetS-associated cognitive disorders.

### 6.2 Therapeutic strategies

Conventional treatments have focused on neuron-focused mechanistic interventions, but drug development and clinical translation have been limited. It is currently considered effective in improving the homeostasis of synapses and their adjacent microenvironments through systems biology and multi-targeted therapeutic approaches. The supportive role and pathologic criticality of neuroglia for neuronal function are gradually being demonstrated. Astrocytes have been proposed at multiple levels as potential targets for drug development to ameliorate central neuropathy and restore cognitive function (202, 203). Based on the altered functional and molecular pathways in astrocytes in MetS, targeting their biological processes may have therapeutic value.

Astrocytes have a central role in neuroinflammation (204). Several studies have targeted toll-like receptor proteins (TLRs) (205), NF-κB, and the transcription factor NF-E2-related factor 2 (Nrf2) (206) in astrocytes, thereby limiting their cell proliferation and neurotoxicity (207). In addition, the repair of astrocyte TJ protein structures resulted in the amelioration of BBB permeability damage. *In vitro* and *in vivo* studies showed that inhibition of astrocyte Cx43 hemichannel opening prevented astrocyte proliferation (astrogliosis) and improved BBB permeability (208). Clomipramine is a classical tricyclic antidepressant. Epoxomicin is a natural selective proteasome inhibitor and has an anti-inflammatory effect. Both drugs have been attempted as inhibitors of intermediate filament proteins and vimentin associated with astrogliosis, thereby facilitating the limitations on nerve regeneration imposed by persistent, excessive glial proliferation (209).

Astrocytic metabolic plasticity allows astrocytes to act as critical cells in maintaining homeostasis (210), with their glycolytic metabolism and derived metabolites, such as lactate and serine, providing energy support to synapses and maintaining homeostasis of neural excitability (211). Therefore, regulation of astrocyte metabolism has also been recognized as a potential therapeutic target (212). The astrocyte glycolytic metabolite L-lactate and secreted vesicles have also been identified as potential targets in neurological disorders (213). Antidiabetic drugs have been found to improve brain glucose uptake by targeting astrocytes. Metformin, which crosses the BBB, increases glucose consumption and lactate release in astrocytes (214). Metformin treatment normalized GLUT-1 expression in STZ-induced diabetic rats and partially restored hippocampal glucose uptake and transport (215). Notably, glucagon-like peptide-1 (GLP-1) receptor agonists have been shown preclinically in small pilot trials to improve cerebral glucose metabolism and functional connectivity (216, 217). Liraglutide (an analog of GLP-1) improves cognitive function by enhancing astrocyte-promoted aerobic glycolysis and alleviating OXPHOS activation to maintain neuronal support (133).

In addition, promoting the transformation of astrocytes into neurons or other glial cells is a potential but controversial therapeutic modality. Several drug tools for astrocyte-specific delivery have been developed, including pluripotent stem cell therapies (218) and effective viral vectors (219) to control astrocyte-specific gene expression. Some studies have used lineage-tracing strategies to target astrocytes *in vivo* for transformation into neurons (220). Other studies have attempted to reprogram astrocyte lineage cells into oligodendrocyte cells by targeting Sox2 and Sox10. This method could relieve astrocyte glial scarring and promote myelin regeneration of neural axons (221–223).

## 7 Conclusions and future perspectives

MetS causes multiple disorders that worsen in the peripheral circulation and affect the brain, which is considered its target organ for generating metabolic stress damage. As a result, MetS accelerates cognitive decline through the acceleration of neurodegeneration and cerebral circulatory disturbances. Astrocytes change their metabolic and immune phenotypes in response to peripheral metabolic stressors, leading to early compensatory regulation of local neurological microenvironmental homeostasis. However, this compensation is lost when the stressors become too much, leading to worsened neuroinflammation. Astrocytes interact with a wide range of cells in the vascular, neural unit to influence BBB permeability and glial lymphatic system drainage functions and also form structures with neural synapses known as tripartite synapses, which play diverse and complex regulatory roles in neural circuit modulation.

The sensitive metabolic and functional plasticity of astrocytes makes them potential targets for improving the maintenance of brain energy metabolism and sustaining synaptic energy support. Also their cellular markers with specific functional proteins are also being developed as diagnostic markers for cognitive disorders. However, the complex and challenging nature of targeting astrocytes by transgenic techniques still poses a challenge due to the rich diversity of astrocytes and their overlap with other CNS cell genetic lineages (224). Contradictory findings in basic research cast doubt on the transdifferentiation capacity of astrocytes (225, 226). Additionally, there is still caution in clinical development regarding immunogenicity mapping in viral manipulation and the potential off-target risk of transgenic manipulation. Nevertheless, this cell implantation strategy could potentially enable endogenous neuronal regeneration in the future.

## Author contributions

LZ: Writing – original draft, Writing – review & editing. JY-Y: Writing – original draft, Writing – review & editing. LC: Formal Analysis, Investigation, Validation, Writing – review & editing. XP: Data curation, Formal Analysis, Funding acquisition, Methodology, Writing – review & editing. TJ: Funding acquisition, Resources, Validation, Visualization, Writing – review & editing. PY: Software, Writing – review & editing. YR: Funding acquisition, Resources, Software, Visualization, Writing – review & editing. PQ:.

## Glossary

**Table:**

18F-FDG	18F-fluorodeoxyglucose
AMPK	5'-AMP-activated protein kinase
PFKFB3	6-phosphofructose-2-kinase/fructose-2 6-bisphosphatase isoform 3
AGEs	Advanced glycation end products
AGT	Ang II precursor molecule angiotensinogen
ANG-1	Angiopoietin-1
ANG	Angiotensin
ACfp	Astrocyte end-feet foot processes
ACfp	Astrocyte endfoot processes
BBB	Blood-brain barrier
CCL2	C-C motif chemokine ligand 2
CBF	Cerebral blood flow
YKL-40	Chitinase-3-like protein 1
CNTF	Ciliary neurotrophic factor
C4b	Complement 4b
Cx43	Connexin 43
CXCL10	C-X-C motif chemokine ligand 10
Ddr1	Discoidin domain receptor 1
EAAT	Excitatory amino acid transporter protein
EVs	Extracellular vesicles
FAs	Fatty acids
GFAP	Glial fibrillary acidic protein
GLUT1	Glucose transporter type 1
GLAST	Glutamate aspartate transporter
GLT-1	Glutamate transporter subtype 1
GAPDH	Glyceraldehyde 3-phosphate dehydrogenase
HSP70	Heat shock protein 70
HDL	High density lipoprotein
HFD	High fat diet
HIF-1α	hypoxia-inducible factor 1-alpha
HIF-1α	hypoxia-inducible factor 1-alpha
IKK β	Inhibitor kappa B kinase β
IR	Insulin resistance
IGF-1	Insulin-like growth factor-1
IKK β	IκB kinase β
LDHB	Lactate dehydrogenase B-chain
LDs	Lipid droplets
LPS	Lipopolysaccharide
LTP	Long-term potentiation
LDL	Low density lipoprotein
MetS	Metabolic syndrome
MCT	Monocarboxylate transporter
mGluR5	Metabotropic glutamate receptor 5
MAO-B	Monoamine oxidase-B
NPY	Neuropeptide Y
NVU	Neurovascular unit
NAD+	Nicotinamide adenine dinucleotide
NAMPT	Nicotinamide phosphoribosyltransferase
NMDA	N-methyl-D-aspartic acid
NF-κB	Nuclear factor-κB
NF-κB	Nuclear transcription factor kappa B
PAPs	Perisynaptic astrocytes processes
PGC-1α	Peroxisome proliferator-activated receptor gamma coactivator-1 alpha
PPAR	Peroxisome proliferator-activated receptor
Kcnj2	Potassium inwardly rectifying channel subfamily J member 2
POMC	Proopiomelanocortin
PTG	Protein targeting glycogen
PKM	Pyruvate kinase
ROS	Reactive oxygen species
RAGEs	Receptors for advanced glycosylation end products
RAS	Renin-angiotensin system
SHH	Sonic hedgehog
Nrf2	Transcription factor NF-E2-related factor 2
TEER	Transendothelial cell electrical resistance
TRPV4	Transient potential receptor vanilloid 4
VEGF	Vascular endothelial growth factor
